# Liposomal bupivacaine vs. Ropivacaine for wound infiltration on chronic postsurgical pain after video-assisted thoracoscopic lung surgery: protocol for a randomized, double-blind, controlled trial

**DOI:** 10.1080/07853890.2025.2543522

**Published:** 2025-08-04

**Authors:** Han-xue Zhao, Wei Dou, Xin-tong Meng, Yi-fan Shi, Xi-sheng Shan, Fu-hai Ji, Shao-mu Chen, Ke Peng

**Affiliations:** a Department of Anesthesiology, First Affiliated Hospital of Soochow University, Suzhou, China; b Institute of Anesthesiology, Soochow University, Suzhou, China; c Department of Thoracic Surgery, First Affiliated Hospital of Soochow University, Suzhou, China

**Keywords:** Chronic postsurgical pain, postoperative analgesia, video-assisted thoracoscopic surgery, liposomal bupivacaine, ropivacaine

## Abstract

**Introduction:**

Chronic postsurgical pain (CPSP) remains a significant challenge for patients undergoing video-assisted thoracoscopic surgery (VATS), compromising their quality of life and postoperative recovery. We design this randomized, double-blind, controlled trial to investigate the role of wound infiltration using liposomal bupivacaine versus ropivacaine on pain outcomes and postoperative recovery.

**Patients and Methods:**

A total of 180 adult patients scheduled to undergo VATS procedures for lung resection will be enrolled. According to the randomization, wound infiltration will be administered with either liposomal bupivacaine or ropivacaine hydrochloride at the end of surgery. Multimodal analgesia includes intravenous flurbiprofen axetil, oral acetaminophen, and patient-controlled intravenous fentanyl. Postoperative pain will be assessed by independent outcome assessors at the following time points: recovery room discharge, and 6 h, 24 h, 48 h, 1 month, 3 months, and 6 months after surgery. The primary outcome is the incidence of CPSP at 3 months. Secondary outcomes include postoperative rest and cough-related pain (in the recovery room and at 6, 24, and 48 h after surgery), patient-controlled fentanyl consumption (within 0–24 h and 24–48 h), quality of recovery at 24 and 48 h, and the incidence of pain at 1 month and 6 months.

**Discussion:**

Our findings will provide evidence on the use of liposomal bupivacaine for postoperative analgesia in patients undergoing VATS lung procedures. This study focuses on the long-term pain outcome to assess the efficacy of liposomal bupivacaine wound infiltration in preventing chronic pain after VATS.

**Trial registration:**

Chinese Clinical Trial Register (ChiCTR2400091157)

## Introduction

Chronic postsurgical pain (CPSP) is a significant issue for patients undergoing thoracic surgery, affecting their postoperative recovery and quality of life. CPSP is defined as pain that develops or intensifies following surgery and persists for at least 3 months [1,2]. Approximately 15–60% of patients undergoing video-assisted thoracoscopic surgery (VATS) experience CPSP [3–6]. Risk factors include younger age, female sex, genetic predisposition, preexisting pain, preoperative opioid use, duration and type of surgery, and acute postoperative pain [4–6]. Among these, inadequate control of acute pain in the early postoperative period is a significant risk factor, and effective management of acute postsurgical pain can minimize CPSP [4]. Multimodal analgesia, which includes opioids, acetaminophen, nonsteroidal anti-inflammatory drugs, regional blocks or local anesthetic infiltration, and other medications, can optimize pain management [7,8]. This approach aims to reduce opioid consumption and associated side effects while improving patient comfort and recovery.

Wound infiltration with local anesthetics such as bupivacaine or ropivacaine is easy to perform for postoperative pain control, providing analgesia for 6 to 12 h [9]. Liposomal bupivacaine is a novel, long-acting, and sustained-release local anesthetic agent. After tissue infiltration, liposomal bupivacaine exhibits a sustained release over 72 h and a biphasic blood concentration profile [10]. It provides analgesia for up to 72 h with minimal side effects and is a promising option for postoperative multimodal analgesia [10]. Some studies have shown that the long-acting analgesic effect of liposomal bupivacaine contributed to better management of postoperative acute pain, thereby reducing CPSP [11–13]. However, others studies have argued that clinical analgesia using liposomal bupivacaine may not exceed that using conventional bupivacaine or ropivacaine [14–16].

Given the lack of evidence, we aim to evaluate the impact of liposomal bupivacaine vs. ropivacaine for wound infiltration on postoperative pain and recovery in patients undergoing VATS lung resection. The primary hypothesis is that liposomal bupivacaine, with its prolonged duration of analgesia, will reduce the incidence of CPSP. We will also investigate acute rest and cough-related pain, patient-controlled fentanyl consumption, and recovery quality after surgery.

### Patients and methods

**Figure 1.**
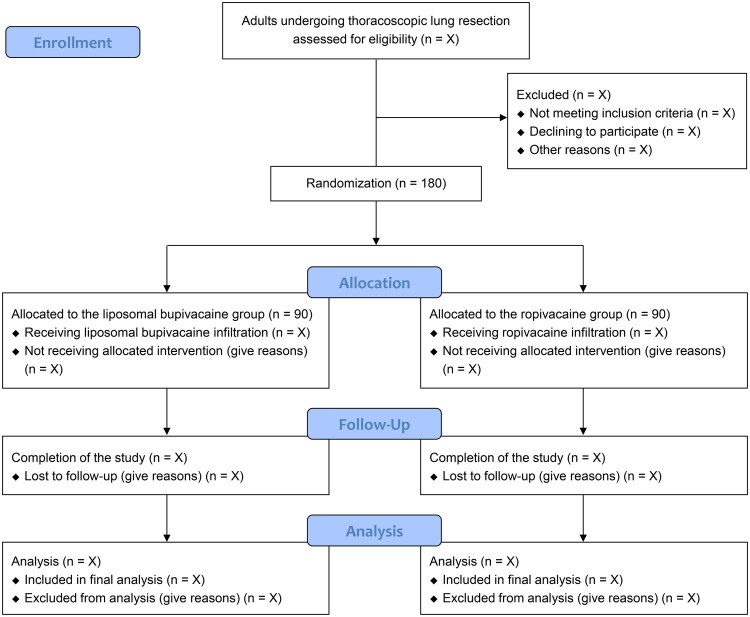
Study flow diagram.

This is a randomized, double-blind, parallel-group controlled clinical trial with a superiority design. We obtained approval by the institutional Ethics Committee (No. 2024-414) on September 30, 2024. We registered the protocol at the Chinese Clinical Trial Registry on October 22, 2024 (ChiCTR2400091157). We plan to recruit 180 eligible patients between October 2024 and November 2025 (*n* = 90 in each group) (Figure 1). All patients will provide informed consent. We will adhere to the Declaration of Helsinki throughout implementation. We report this protocol based on the statement of the Standard Protocol Items: Recommendations for Interventional Trials (SPIRIT) (Supplement 1) [17].

#### Patients

The inclusion criteria are as follows: (1) adult patients age ≥ 18 years old, (2) ASA classifications I–III, and (3) scheduled to undergo VATS procedures for lung resection. Patients will be excluded if they meet any of these criteria: (1) allergy to local anesthetics (such as bupivacaine, ropivacaine, or lidocaine), (2) history of previous thoracic surgery, (3) neuropathic or chronic pain (persistent or recurrent pain for > 3 months), (4) long-term use of analgesics (analgesic use for > 3 months), or (5) inability to communicate effectively or refusal to participate.

#### Randomization and blinding

An independent researcher generates a randomization list using the Sealed Envelope tool with a ratio of 1:1 and blocks of 4, 6, and 8. To ensure allocation concealment, the list will be stored in sealed opaque envelopes. After anesthesia induction, an independent research nurse will access the random results and assign patients to either the liposomal bupivacaine group or the ropivacaine group (Figure 1). This nurse will also prepare the study drugs in identical opaque syringes in a sterile manner and deliver them to surgeons for administration. All patients, clinicians, and postoperative outcome assessors will be fully masked to the allocation. It is still possible that the surgeons may distinguish the anesthetics when performing the injections; however, the hypothesis will not be disclosed.

#### Anesthesia management

General anesthesia will be induced using i.v. dexamethasone 5 mg, sufentanil 0.3 μg/kg, and propofol 1.5–2 mg/kg. After rocuronium 0.8 mg/kg is administered, trachea is intubated with a double-lumen tube followed by one-lung ventilation as needed. Anesthesia will be maintained with sevoflurane, targeting the bispectral index of 40–60. Palonosetron 0.25 mg will be given. Hemodynamic events (hypotension [decrease in mean blood pressure > 30% from baseline], hypertension [increase in mean blood pressure > 30% from baseline], bradycardia [heart rate < 50 beats/min], and tachycardia [heart rate > 50 beats/min]), fluid infusion, and other anesthetic care will be managed by attending anesthesiologists. Patients will be extubated in the recovery room. A standardized Enhanced Recovery After Surgery (ERAS) protocol will be applied [18–20].

Postoperative multimodal analgesia includes (1) wound infiltration with liposomal bupivacaine or ropivacaine at the end of surgery; (2) oral acetaminophen 500 mg every 6 h; (3) flurbiprofen axetil 50 mg twice daily within the first two postoperative days; and (4) patient-controlled i.v. analgesia (PCA) with fentanyl (a 10-µg bolus dose at a 5-min lockout interval) for 48 h postoperatively. Patients will rate pain intensity using the numeric rating scale (NRS), which ranges from 0 (indicating no pain) to 10 (indicating the more severe pain). Patients will be informed to self-administer PCA boluses to relieve significant postoperative pain (NRS scores ≥ 4).

#### Study interventions

According to the randomization, patients will receive wound infiltration with liposomal bupivacaine (*n* = 90) or ropivacaine (*n* = 90). At the end of surgery and before skin closure, surgeons will perform would infiltration using the corresponding local anesthetics within 5 min (multilayered administration from the subcutaneous tissue to the parietal pleura). The liposomal bupivacaine group will receive wound infiltration with liposomal bupivacaine 266 mg (3–5 ml for each 10-mm port site; diluted with normal saline if needed), while the ropivacaine group will receive 0.375% ropivacaine (3–5 ml for each 10-mm port site).

#### Primary and secondary outcomes

Postoperative NRS pain scores will be assessed at recovery room discharge and 6 h, 24 h, 48 h, 1 month, 3 months, and 6 months postoperatively. The primary outcome is the incidence of CPSP at 3 months postoperatively (defined as pain with NRS scores ≥ 1) [21]. Secondary outcomes are (1) rest and cough NRS pain scores in the recovery and at 6, 24, and 48 h postoperatively; (2) consumption of PCA fentanyl within 0–24 h and 24–48 h; (3) recovery quality at 24 h and 48 h postoperatively, measured with the 15-item quality of recovery (QoR-15) scale (a total score of 150 and a minimal clinically important difference of 6) [19,22,23]; and (4) the incidence of pain at 1 month and 6 months postoperatively.

#### Data collection

Table 1 presents the schedule of enrollment, interventions, and data measurement following the SPIRIT guideline. Demographic data, ASA status, comorbidities, preoperative medications, and age-adjusted Charlson Comorbidity Index (aCCI) will be collected pre-anesthesia. Surgical, anesthesia, and perioperative data (including hemodynamic events) will be collected on an electronic system (Suzhou MedicalSystem DoCare V5.0) [24]. The following data will be extracted using the electronic records: postoperative hospital stay and complications (myocardial ischemia or infarction, new-onset arrythmias, heart failure, pneumonia, pulmonary edema, respiratory failure, stroke, renal failure, need for dialysis, sepsis, postoperative hemorrhage, unplanned admission to the intensive care unit, and reoperation).

**Table 1.** Schedule of patient enrollment, study interventions, and outcome assessment according to SPIRIT statement.

			Study period							
	Enrollment	Allocation	Post-allocation							Closeout
Timepoint	Preoperative visit	Before surgery	During surgery	Recovery room	6 h	24 h	48 h	1 month	3 months	6 months
**Enrollment**										
Inclusion criteria	**×**									
Exclusion criteria	**×**									
Written informed consent	**×**									
Baseline data	**×**									
Randomization		**×**								
Allocation		**×**								
**Study interventions**										
Liposomal bupivacaine			**×**							
Ropivacaine			**×**							
**Outcome assessment**										
Pain at rest				**×**	**×**	**×**	**×**	**×**	**×**	**×**
Pain on coughing				**×**	**×**	**×**	**×**	**×**	**×**	**×**
Patient-controlled fentanyl						**×**	**×**			
Quality of recovery						**×**	**×**			

During the postoperative follow-up, assessors who are unaware of assignment details will document the study outcomes (including pain intensity, PCA fentanyl consumption, and quality of recovery) and any adverse effects associated with local anesthetics (such as symptoms of local anesthetic toxicity). The study implementation will be monitored by an independent data monitoring committee (consisting of an attending thoracic surgeon, an attending anesthesiologist, a pharmacist, and a statistician).

#### Sample size estimation

Based on previous studies, the incidence of CPSP following VATS ranged from 15% to 60% [3,4,25,26]. We hypothesize a CPSP rate of 40% with ropivacaine and 20% with liposomal bupivacaine. To test this difference with a two-sided significance level = 0.05 and a power = 80%, 80 patients in each group are required. Accounting for a 10% dropout rate, recruitment of a total of 180 patients is planned (PASS 11, NCSS, USA).

#### Statistical analysis

The Shapiro-Wilk test will be used to assess data normality, with mean ± standard deviation presented for normally distributed data or median (interquartile ranges) for skewed data. Categorical data will be expressed as n (%). For the pain outcomes at 1 month, 3 months, and 6 months after surgery, Chi-square test or Fisher’s exact test will be applied to compare the two groups. The other outcomes (NRS pain scores, patient-controlled fentanyl consumption, and QoR-15 scores) will be analyzed using unpaired t test or Mann-Whitney test, as appropriate. To further analyze the effect size of study interventions, relative risk or difference in means or medians and their 95% confidence intervals will be calculated. In addition, we will perform a prespecified subgroup analysis according to patient age, sex, aCCI, and acute postoperative pain control.

Data will be analyzed in all randomized patients who undergo VATS procedures with available outcome data. Missing data will not be imputed. No interim analysis is planned. The significance level for the primary outcome is a two-sided *p* < 0.05. For the secondary outcomes, Benjamini-Hochberg multiple testing adjustment will be applied, with a significance level of false discovery rate *q* < 0.05. Statistical analyses will be conducted using the SPSS 25.0 software (IBM SPSS, Chicago, IL, USA)

## Discussion

This randomized clinical trial will include 180 adults having VATS procedures to determine whether wound infiltration with liposomal bupivacaine, compared with ropivacaine hydrochloride, would reduce the rate of CPSP. Rest and cough-related pain intensity, PCA fentanyl consumption, quality of recovery after surgery, and the incidence of pain up to 6 months postoperatively are among the secondary outcomes. The guidelines of Consolidated Standards of Reporting Trials will be followed [27].

CPSP is often localized to the area of injury or may radiate to areas innervated by the affected nerves, particularly after deep somatic or visceral tissue damage. We adopt the 3-month incidence of CPSP as the primary outcome according to its definition, and we will perform an extended follow-up at 6 months. Risk factors of CPSP after thoracic surgery include injury to intercostal nerves during surgical procedures, infections, pleural inflammation, and poor acute pain control in the early postoperative period [28]. Multimodal analgesia strategies can optimize pain relief and reduce opioid-related adverse effects. Epidural analgesia, paravertebral block, intercostal nerve block, erector spinae plane block, and wound infiltration are among the components of multimodal analgesia in thoracic surgery [29–32]. Recently, we have applied wound infiltration with ropivacaine for pain control in VATS procedures [20,33].

Dr. Kjærgaard and colleagues performed a systematic review to suggest that wound infiltration with local anesthetics led to small or modest reductions in pain intensity, mainly immediately after surgery [34]. A recent review updates the use of wound infiltration with local anesthetics for postoperative pain management [35]. The benefits of wound infiltration have been documented in open and minimally invasive surgeries. Dr. Huang and colleagues conducted a meta-analysis of randomized controlled trials to conclude that continuous wound infiltration exerts effective postoperative analgesia with a good safety profile and is a promising analgesic option to enhance recovery after laparotomy and sternotomy procedures [36].

Studies have investigated liposomal bupivacaine for pain management following procedures involving the trunk or extremities. Interscalene block using liposomal bupivacaine was compared with a combination of bupivacaine, dexamethasone, and epinephrine in shoulder replacement, showing that postoperative pain scores within 24–48 h, 48–72 h, 72–96 h, and at day 60 after surgery were significantly lower in the liposomal bupivacaine group [12]. Dr. Bauerle and colleagues showed that intercostal nerve blocks with liposomal bupivacaine as part of an ERAS protocol for VATS lobectomy was associated with a reduced opioid use and a shorter length of hospitalization [11]. Dr. Banks et al. observed a relationship between liposomal bupivacaine and reductions in opiate utilization and hospital stay [37]. In patients undergoing VATS and thoracotomy, paravertebral block with liposomal bupivacaine provided effective pain management [38]. However, the superiority of liposomal bupivacaine over plain bupivacaine has been challenged. A study found no significant difference in pain outcomes after shoulder replacement between liposomal bupivacaine and plain bupivacaine [15]. A recent meta-analysis suggested that perineural liposomal bupivacaine improved analgesia after orthopedic surgery in a statistically but not clinically significant manner [39]. Another meta-analysis showed low-level evidence that liposomal bupivacaine in brachial plexus block may reduce pain after upper limb surgery, with a limited clinical significance [40]. Nonetheless, the effects of liposomal bupivacaine vs. ropivacaine on CPSP and postoperative recovery following VATS lung resection are yet to be determined.

The application of ERAS is especially important for VATS procedures [41]. ERAS is a set of perioperative protocols, aiming at enhancing recovery, improving surgical outcomes, and reducing hospital stays [42]. At our institution, the ERAS protocol has been well integrated into the perioperative care of VATS procedures [18]. Our recent studies have also adopted this protocol to reduce adverse events and improve the quality of recovery [19,20]. By conducting this study, we expect to refine the ERAS protocol and optimize personalized care in this patient population.

The limitations of our study include: (1) liposomal bupivacaine will be utilized exclusively for incisional infiltration in our patients. Its potential benefits in other regional analgesia techniques (such as paravertebral block and erector spinae plane block) warrant further explorations; (2) CPSP is closely associated with psychological disorders such as anxiety and depression, which are not assessed in this study; and (3) this study will be conducted at a single center in China. Broader validations by multicenter trials are necessary to confirm the findings and enhance generalizability.

In conclusion, this study will primarily assess the impact of liposomal bupivacaine vs. ropivacaine hydrochloride for local infiltration analgesia on the incidence of CPSP in adult patients undergoing VATS lung resection procedures. By conducting this study, we expect to refine multimodal analgesic strategies and enhance recovery for these patients.

## Supplementary Material

Supplement.doc

## Data Availability

The full protocol, participant-level dataset, statistical plan, and informed consent materials can be available *via* contacting the corresponding author after the formal publication of this trial.
